# Métastases mammaires d’un adénocarcinome gastrique associées à une grossesse de 32 semaines d’aménorrhée: à propos d’un cas rare

**DOI:** 10.11604/pamj.2013.15.116.2922

**Published:** 2013-07-31

**Authors:** Sofia Jayi, Hind Fatemi, Kamilia Laabadi, Hakima Bouguern, Hikmat Chaara, Afaf Laamarti, My Abdelilah Melhouf

**Affiliations:** 1 Université Sidi Mohammed Benabdellah, Service de Gynéco-Obstétrique 2, CHU Hassan II, Fès, Maroc; 2 Université Sidi Mohammed Benabdellah, Service d’anatomopathologie, CHU Hassan II, Fès, Maroc

**Keywords:** Métastase mammaire, cancer gastrique, diagnostic, grossesse, breast metastasis, gastric cancer, diagnosis, pregnancy

## Abstract

Les métastases mammaires ne représentent que 0,4 à 2% des tumeurs malignes du sein. L’origine gastrique est exceptionnellement rapportée. Encore plus rare est l’association des métastases mammaires et ovariennes provenant de l’estomac. De par leur rareté et la non spécificité des signes cliniques et radiologiques, les métastases mammaires peuvent être confondues avec un cancer primitif et être à l’origine d’un traitement mutilant non approprié. Nous rapportons le cas d’un adénocarcinome gastrique métastatique au niveau mammaire et ovarien chez une patiente jeune de 32 ans et enceinte de 32 Semaines d’aménorrhée, qui est à notre connaissance le premier cas rapporté au cours de la grossesse. A travers notre cas, nous soulignons les caractéristiques épidémiologiques, diagnostiques, thérapeutiques et pronostiques de cette entité très rare, dont la connaissance est nécessaire afin d’éviter les erreurs diagnostic et les prise en charge non adaptée qui peut suivre.

## Introduction

Les métastases mammaires sont rares, elles représentent 0,4 à 2% de l’ensemble des tumeurs malignes du sein. L’origine gastrique est exceptionnellement rapportée. Encore plus rare, est l’association synchrone ou métachrone des métastases mammaires et ovariennes provenant de l’estomac [1, 2]. Nous rapportons le cas d’un adénocarcinome gastrique métastatique au niveau mammaire et ovarien chez une patiente enceinte de 32 SA, qui est à notre connaissance le premier cas rapporté au cours de la grossesse.

## Patient et observation

**Figure 1:**
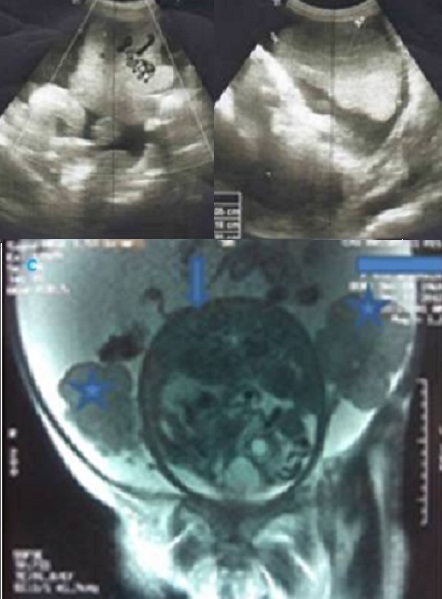
Echographie montrant les masses ovariennes bilatérales droite (A) et gauche (B), évoquant l’origine métastatique; C: IRM montrant les masses ovariennes (étoiles), et l’utérus gravide contenant la grossesse (flèche)

**Figure 2:**
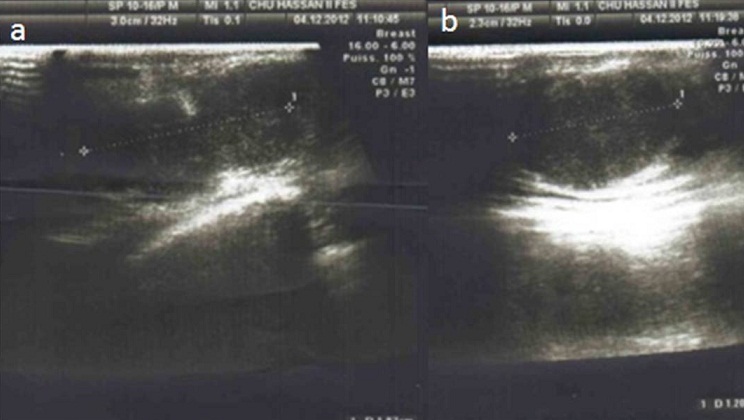
Aspect échographique des 2 lésions mammaires palpables (A: sein droit, B: sein gauche) classées ACR 4

**Figure 3:**
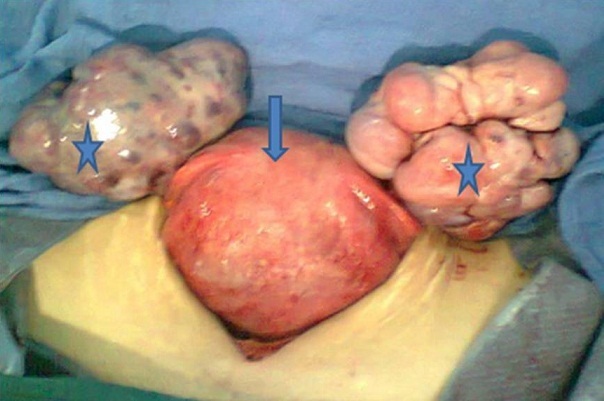
Image per opératoire montrant l’utérus après extraction fœtale (flèche) et l’aspect tumorale des 2 ovaires (étoiles)

**Figure 4:**
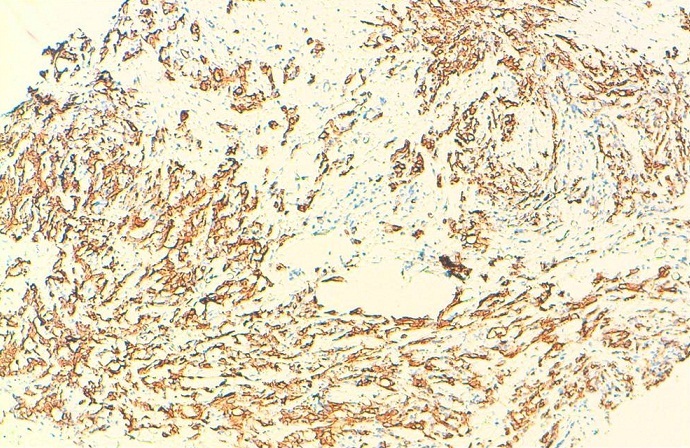
Les cellules tumorales CK20+ infiltrant le parenchyme mammaire

**Figure 5:**
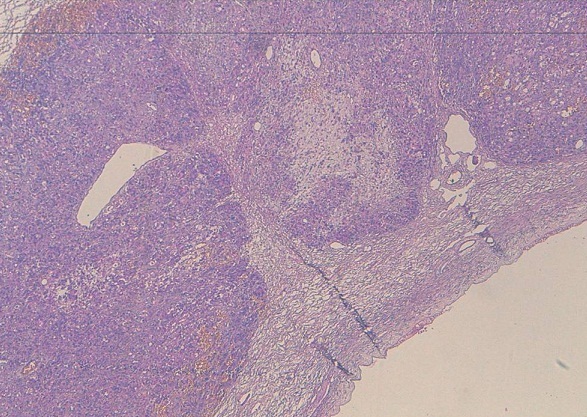
Prolifération carcinomateuse peu différenciée qui se dispose en nappes diffuses infiltrant le parenchyme ovarien (HESx5)

**Figure 6:**
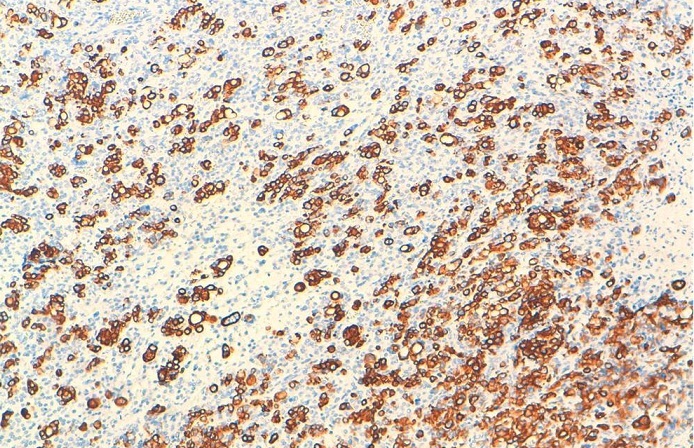
Positivité diffuse des cellules tumorales avec l’anticorps anti CK 20

**Figure 7:**
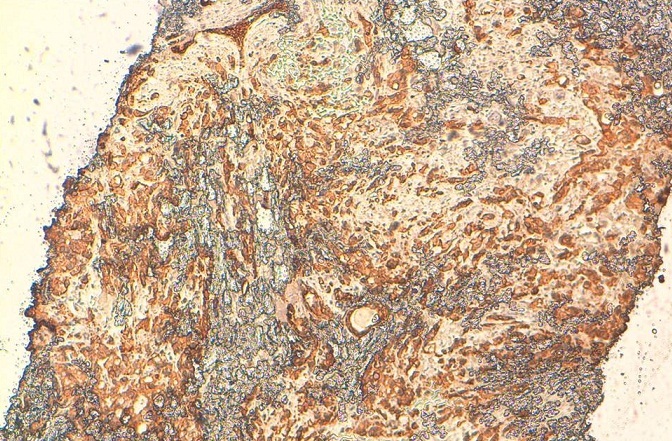
La positivité des cellules tumorales avec l’anticorps anti CK7

Mme S.A. âgée de 32 ans, sans antécédent pathologique connu, G6P5, grossesse actuelle non suivie estimée à 32 semaines d’aménorrhée, ayant consulté pour distension abdominale importante avec altération de l’état général. L’examen clinique trouve une patiente cachectique, abdomen très distendu. L’examen des sein a trouvé 2 nodules (l’ un au niveau de la jonction des quadrans inferieurs du sein droit faisant 2cm et l’ autre au niveau du quadran supéro interne du sein gauche faisant 1 cm) à contours polylobés, de consistance ferme et mobiles par rapport au 2 plans, avec des adénopathies axillaires gauches et susclaviculaires. Le toucher rectal a objectivé un aspect induré de la face antérieure du rectum. L’exploration pelvienne par l’échographie complétée par l’IRM (Figure 1) a trouvé une grossesse intra-utérine évolutive de 32 SA, avec une ascite de grande abondance et 2 tumeurs latéro-utérines droite et gauches mesurant respectivement 86 cm et 98cm de grand axe, évoquant une tumeur de krukenberg bilatérale avec des signes de carcinose péritonéale qui au niveau du douglas, se continue avec l’induration rectale. L’origine rectale a été évoquée d’autant plus que la patiente n’a pas rapporté de signe digestif haut. Cependant, la biopsie rectale s’est révélée négative. Par ailleurs, L’échographie hépatique était normale et la TDM thoracique a trouvé des lésions ostéo-condensantes en faveur de localisations secondaires avec sur la NFS une bicytopénie. L’échographie mammaire a classé les nodules palpables ACR 4 (Figure 2) et a objectivé 2 autres lésions infra-cliniques droites (6 et 7 mm) classées ACR 4. Après discussion du cas en réunion de concertation multidisciplinaire, on a réalisé une césarienne (après corticothérapie pour maturation pulmonaire fœtale) avec une annexectomie diagnostique bilatérale (Figure 3). L’exploration per opératoire a trouvé un nodule péritonéal du douglas, un épiploon d’allure tumorale, un estomac tumoral et le geste a été complété par des biopsies multiples. A la fin de l’intervention, une biopsie des 2 nodules mammaires palpables a été réalisée. L’étude histologique a conclue à une localisation mammaire (Figure 4), ovarienne (Figure 5, Figure 6, Figure 7), péritonéale, et épiploique, d’un adénocarcinome digestif dont les cellules expriment franchement le CK20 et faiblement le CK7 à noter que celles d’origine mammaires n’expriment pas les récepteurs oestrogéniques et progestéroniques avec un témoin interne négatif.

A J 3 de la césarienne, la fibroscopie avec biopsie de la tumeur a été faite. Et l’histologie du processus gastrique a trouvé un adénocarcinome gastrique peu différencié dont les cellules expriment la CK et n’expriment pas le CD20. Cependant la patiente est sortie contre avis médical quelques jours après la césarienne.

## Discussion

Les tumeurs métastatiques du sein sont très rares et représentent 0,5 à 6% de tous les cancers du sein [3]. Les tumeurs primitives les plus fréquemment rapportées chez les adultes sont les lymphomes, les leucémies, les mélanomes, suivies par les tumeurs pulmonaires, ovariennes et rénales; chez l’enfant [1–3], alors que les Lésions gastro-intestinales ne métastasent que rarement au niveau des seins [3]. Elles sont souvent diagnostiquées dans un contexte poly-métastatique comme c’est le cas de notre patiente qui a présenté des métastases ostéo-médullaires compliquées de bicytopénie, ovariennes, péritonéales, et épiploiques. Cependant, l’association des métastases mammaires et ovariennes - métachrones ou synchrones - d’un cancer gastrique sont exceptionnelles [1]. En effet, Le premier cas de métastases mammaires et ovariennes de carcinome à cellules en bague à chaton gastrique a été rapporté dans la littérature en 1999 et depuis, seulement 5 cas ont été signalés [3], ce qui confirme la rareté de notre cas. L’intervalle de temps séparant le diagnostic de la métastase mammaire et de la tumeur primitive est variable selon les auteurs et peut aller jusqu’à 8 ans mais elles peuvent dans certains cas comme celui de notre patiente être révélatrices de la tumeur primitive [1–4]. L’âge moyen des patientes présentant des métastases mammaires d’origine gastrique est estimé à 47 ans [1, 3, 5].

En effet, la localisation secondaire au niveau du sein qui est un organe hormono-dépendant semble assez intrigante, en particulier chez ces femmes non ménopausées et jeunes - comme c’est le cas de notre patiente qui est âgée de 32 ans. La richesse de la vascularisation du sein chez la femme en activité génitale a été avancée pour expliquer l’atteinte mammaire chez cette catégorie. En outre, le cancer gastrique semble avoir un comportement biologique plus agressif dans les groupes d′âge plus jeune, où les facteurs hormonaux sont impliqués [3]. Ainsi, L’implication hormonale serait encore plus importante en cas de grossesse ce qui pourrait majorer davantage l’agressivité de ces tumeurs.

Concernant la localisation mammaire. Les formes multifocales et bilatérales sont plus rares et l’aspect clinique et radiologique est volontiers trompeur et peut simuler un cancer mammaire primitif [4–6]. Cliniquement, il s’agit d’une tumeur palpable, localisée fréquemment dans le quadrant supéro-externe du sein gauche, les formes multifocales et bilatérales - comme c’est le cas de notre patiente- sont plus rares (25%) [1–3]. Classiquement, il n’existe pas de modification cutanée ni mamelonnaire [1], occasionnellement, des formes simulant un cancer inflammatoire du sein avec œdème et rougeur cutanée ont été rapportées et la présence d’adénopathie axillaire est notée dans plus que 15% des cas [3].

Sur la mammographie, les lésions métastatiques peuvent apparaître comme des lésions bénignes, et des masses circonscrites sans microcalcifications. L’aspect spiculé est classiquement absent, en rapport avec une réaction desmoplastique minime du stroma dans les lésions métastatiques [3] (La mammographie n’a pas été réalisée chez notre patiente vue qu’elle était enceinte).

Histologiquement, les métastases mammaires posent un problème de différenciation avec l’adénocarcinome primitif mammaire et pour les cellules en bague à chaton avec le carcinome lobulaire à cellules en bague à chaton. La reconnaissance des tumeurs métastatiques du sein est d’une importance capitale car elle permettrait d′éviter une chirurgie mutilante inutile et conduirait à un traitement approprié de la tumeur Primitive [4]. Pour cela, l’immunohistochimie se révèle d’un grand intérêt. En effet la positivité des cellules pour l’ACE et la CK20 et la négativité des récepteurs oestrogéniques et progesteroniques et de c-erb-B2 (dans un maximum de 20%, il peut être positif) [3] plaident en faveur de l’origine gastrique d’autant plus qu’on a une métastase ganglionnaire sus-claviculaire [1–7]. Ainsi, dans notre cas la positivité de la CK 20, la négativité des récepteurs oestrogéniques et progestéroniques au niveau des cellules tumorales mammaires, en plus de la présence de l’adénopathie sus-claviculaire, étaient des arguments forts en faveur de l’origine gastrique.

La chirurgie loco-régionale carcinologique et la radiothérapie sont loin d′être le traitement approprié pour les métastases mammaires d’origine gastrique [3–8]. La chirurgie est indiquée pour le contrôle local de la tumeur, couplé à une thérapeutique systémique [1].

Les métastases du sein d’un cancer primaire gastro-intestinal ont un mauvais pronostic, en particulier ceux d′origine gastrique [3, 8, 9] puisque 80% des patientes environ, décèdent un an après le diagnostic [3–8].

## Conclusion

Il est admis que chez jusqu′à 25% des patientes ayant des métastases mammaires, la lésion primaire n′a pas encore été diagnostiquée, et la masse mammaire palpable pourrait être le premier signe d′une maladie inconnue [3]. Pour cela, nous en tant que praticiens, nous devons garder à l’esprit que la métastase mammaire peut simuler cliniquement et radiologiquement le cancer primitif, et que le diagnostic différentiel - basé sur l’histologie et appuyé par l’immunohistochimie- est primordial afin d’éviter aux patientes une chirurgie mutilante inutile et de leur offrir une prise en charge adaptée à la tumeur primitive.
